# Weakening of the Active Muscle Restraint Against Valgus Loading During Repeated Baseball Pitching: Direct Evidence of Fatigue in Dynamic Valgus Stabilizers of the Elbow Joint in Collegiate Baseball Pitchers

**DOI:** 10.1177/03635465261460711

**Published:** 2026-07-06

**Authors:** Toshimasa Yanai, Kengo Onuma

**Affiliations:** † Faculty of Sport Sciences, Waseda University, Tokorozawa, Japan; ‡ Research Institute of Baseball Science, Waseda University, Tokyo, Japan; Investigation performed at Waseda University, Tokorozawa, Japan

**Keywords:** biomechanics, flexor-pronator muscle mass, inverse dynamics, ligamentous injury, throwing

## Abstract

**Background:**

Although research has shown the effect of repeated pitching on throwing arm strength, no studies have examined the weakening in the overall ability of dynamic stabilizers to restrain the medial elbow against valgus loading. Fatigue in the dynamic stabilizers increases reliance on the ulnar collateral ligament (UCL) to withstand the valgus loads during pitching.

**Hypothesis:**

It was hypothesized that (1) the muscular varus strength weakens as the pitch count accumulates and (2) the muscular varus strength becomes insufficient to overcome the valgus loads in later innings.

**Study Design:**

Descriptive laboratory study.

**Methods:**

A total of 28 collegiate baseball pitchers threw 7 innings of 15 pitches in a bullpen. Seven genlocked cameras were used to record pitching performances, and the elbow valgus load was determined. Muscular varus strength was measured before the first inning and after completing the fourth and seventh innings using an isokinetic dynamometer system and an ultrasound device. A mixed-effect model was used to examine changes over the innings in the valgus load and varus strength, and *t* tests were used to compare the varus strengths and the largest valgus load in corresponding innings.

**Results:**

Neither the largest valgus load of the inning nor the mean valgus load of the inning significantly changed across the innings, whereas the varus strength was reduced significantly after completing the fourth and seventh innings (*P* < .001). The varus strength was significantly greater than the mean valgus load in the first inning (*P* < .008) but not in the later innings.

**Conclusion/Clinical Relevance:**

Pitchers’ medial elbow muscular varus strength decreased by a mean of 10% after 7 innings of 15 pitches, while motion-dependent valgus load remained fairly constant throughout the 7 innings, which could indicate increased reliance on the UCL to withstand the valgus loads in later innings.

The ulnar collateral ligament (UCL) is one of the most affected sites of overuse injuries to baseball pitchers. Literature on Major League Baseball pitchers has reported that elbow injuries account for approximately 30% of all baseball-related injuries,^32^ and the so-called Tommy John surgery, a surgical technique for reconstructing damaged UCL,^19^ has been performed more frequently in the present decade.^8,18,21^ Such a high risk of UCL injury among baseball pitchers is attributed to the inability of the UCL to withstand repeated valgus stress, which opens the inner elbow compartment^24^ and causes elongation of the ligament that over time can lead to attenuation and failure.^34^

During baseball pitching, elbow structures generate varus moments of up to 50 to 120 N·m at or near the end of the arm cocking phase.^6,13-15,33,38,39^ This varus moment functions to resist the motion-dependent valgus loads acting on the elbow joint and to restrain valgus angulation against valgus overloads (Figure 1). The varus moment is produced not only by a combination of tensile forces of the UCL and joint capsule and compressive forces on the bones of the lateral side of the elbow joint,^24,25^ but also by contractile forces of the medial elbow musculature^5,9,17,20,25,35^ such as the flexor carpi ulnaris, flexor digitorum superficialis, and pronator teres muscles. Therefore, unlike the case in cadaveric studies where any amount of valgus loading results in elongation of the UCL, valgus loads applied to the elbow joint during pitching do not necessarily open the medial elbow compartment and elongate the UCL due to synergistic contractions of medial elbow musculature.^10,16,43^

**Figure 1.**
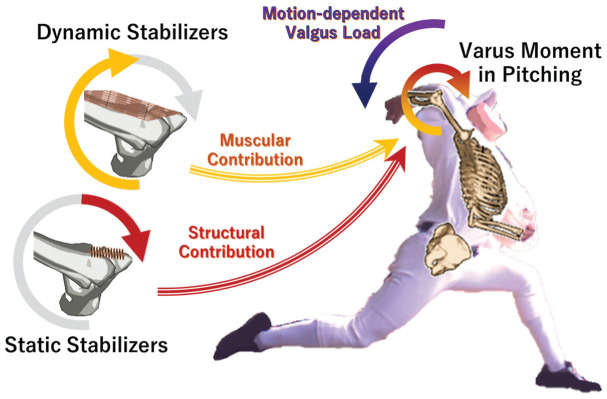
Varus moment in pitching and its contributions. Varus moment functions to resist the motion-dependent valgus loads acting on the elbow joint and to restrain valgus angulation against valgus overloads. The varus moment is produced by static stabilizers (eg, ulnar collateral ligament, joint capsule, and bony articulation) and dynamic stabilizers (eg, flexor carpi ulnaris, flexor digitorum superficialis, and pronator teres).

The strength of the medial elbow musculature in resisting valgus loading has been measured in recent studies.^42,43^ A case report of 2 professional baseball pitchers^42^ who had just returned to pitching in simulated game situations after UCL reconstruction surgery found that their medial elbow musculatures were capable of generating isometric varus moments of 71 N·m and 59 N·m, respectively, with maximal effort. Another experimental study^43^ reported that for 22 healthy professional and collegiate pitchers, the mean muscular varus strength of the medial elbow musculature was 57.5 ± 9.2 N·m, which was far greater than the varus moment that the UCL and other passive structures of the elbow joint could generate before failure.^4,7,22^ Based on the electromyographic (EMG) studies showing that the elbow flexor-pronator muscles are highly active during the arm cocking and acceleration phases of the pitching motion,^10,16^ the aforementioned observations suggest that the medial elbow musculature should produce a substantial amount of varus moment to act against the valgus loads during baseball pitching. These observations raise a question: if the medial elbow muscles could generate substantial varus moment to protect the UCL from valgus overloads, how come many pitchers sustain valgus overload injuries?

One possible explanation is the effect of muscle fatigue. Starting pitchers often throw ≥100 pitches per game, which may lead to muscle fatigue in both the upper and lower bodies. In fact, studies have reported that as the cumulative pitch count reaches 60 to 105, shoulder muscle strength decreases by 10% to 18%,^23,27,31,41^ grip strength decreases by 5% to 12%,^26,28^ ulnar deviation strength decreases by 12%,^28^ pronator strength decreases by 6%,^31^ and finger flexor strength decreases by 7% to 21%.^26^ Weakening of these muscles may change pitching mechanics, weaken the active muscle restraint against valgus loading, and possibly increase the risk of elbow valgus overload injury. However, no studies have directly examined the decline in the overall ability of dynamic stabilizers to resist valgus loads on the elbow. Therefore, the present study was conducted to examine the effects of repetitive pitching on the valgus load applied to the elbow joint and the strength of the medial elbow musculature that resists valgus loading. Two hypotheses were tested: (1) muscular varus strength weakens as the pitch count accumulates, and (2) muscular varus strength becomes insufficient to overcome the valgus loads in later innings.

## Methods

### Data Collection

A total of 28 pitchers of a collegiate baseball team (mean age, 20 ± 0.9 years; mean mass, 78 ± 7.6 kg; mean height, 1.77 ± 0.061 m) participated in this study. Eight participants had experienced shoulder or elbow pain at least once in the past 3 years of their baseball careers, and one of them had undergone UCL reconstruction >1.5 years before this study. All participants were self-declared as healthy and as having no physical injury or pain on the experimental days that would hinder their normal pitching performance. An a priori power analysis using G*Power^12^ showed that a minimum sample size of 17 to 28 participants was required for repeated-measures analysis of variance (across 3-7 sessions), assuming a small effect size (0.25) and a power of 80%; therefore, our sample size was considered sufficient. The study conforms to the Declaration of Helsinki, and the research protocol was approved by our institutional ethics committee. The procedure and risks associated with participating in this study were explained to each participant, and they provided written informed consent.

**Figure 2.**
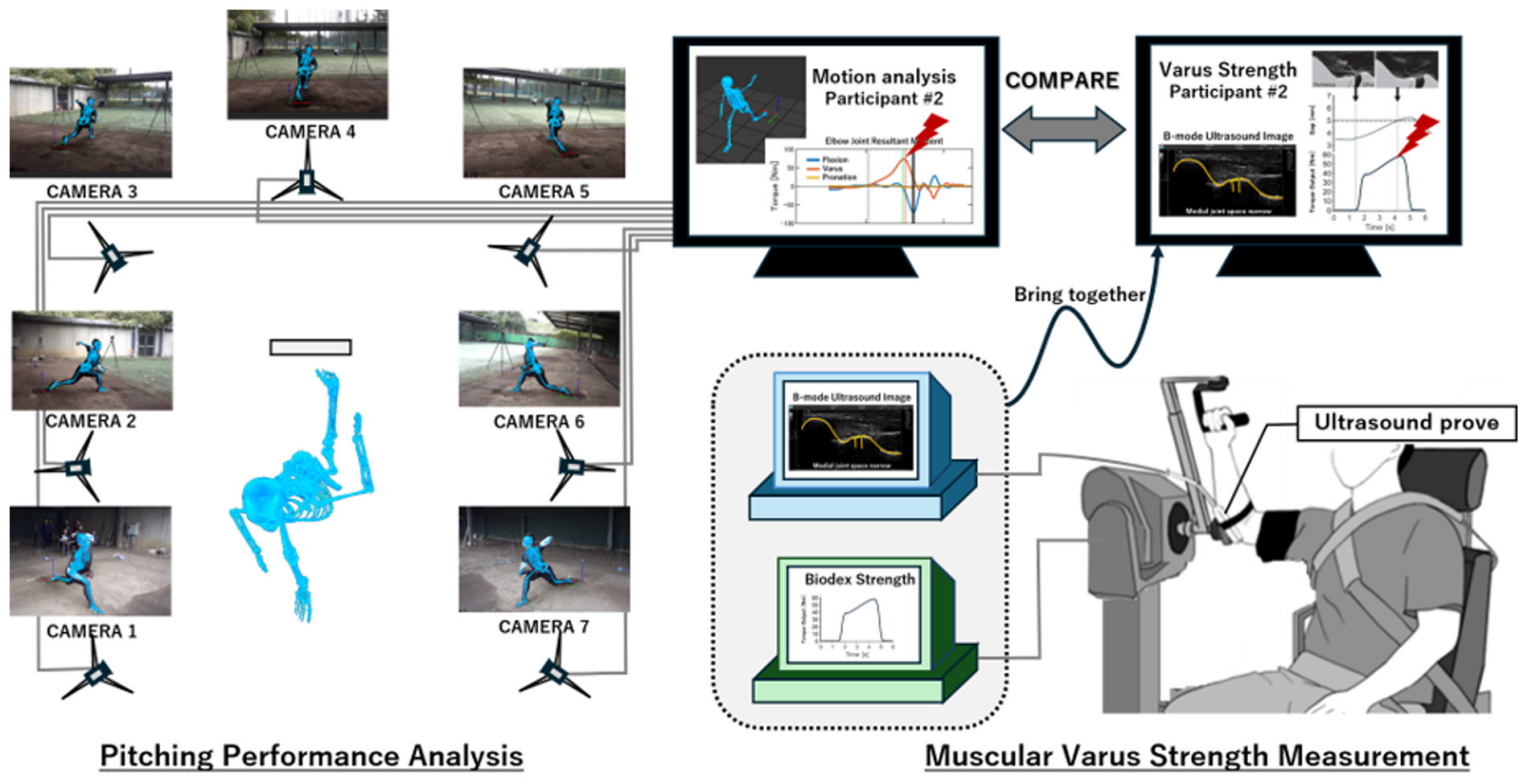
Experimental setup. Seven pairs of genlocked digital cameras and camera control boxes were used to record pitching performances and determine motion-dependent valgus loads. An isokinetic dynamometer system was used to measure the torque output, and, simultaneously, a B-mode ultrasound device genlocked with the dynamometer system was used to measure the width of the medial joint space between the trochlea of the humerus and the sublime tubercle of the ulna. Time-series data of torque output and medial joint space width were synchronized to identify the largest varus moment that each participant generated without the medial joint space expanding beyond a predetermined individualized threshold distance. This largest moment was taken as the maximum voluntary isometric varus strength of the participant's elbow and evaluated against the participant’s valgus load for each pitch.

After a self-determined warm-up (jogging, stretching, shoulder exercises, playing catch, long toss, etc), each participant was asked to throw 7 innings of 15 pitches in a bullpen at a baseball field where the team regularly practices. Wearing metal cleats and practice uniform, they threw all pitch types that they used during games from the pitcher's mound toward a catcher crouched behind home plate, located at the regulation distance from the pitcher's plate (18.44 m or 60 feet 6 inches). The number of pitches of each type and the order in which the available pitch types were to be thrown were not specified, and the participants were asked to throw a combination of all pitch types as in a game situation. There were scheduled 5-minute breaks between innings, with a longer break of up to 15 minutes between the fourth and fifth innings for muscular varus strength testing in a laboratory near the bullpen. This longer break conveniently replicated the practice of taking an approximately 10-minute break for field maintenance during official games in the Tokyo Big6 Baseball League, of which the team is a member. The participants were allowed 5 to 6 warm-up pitches before the start of each inning. The pitching performances were recorded at the sampling frequency of 120 Hz with 7 pairs of genlocked digital cameras (Cyber-shot RX0 II; SONY) and camera control boxes (CCB-WD1; SONY) (Figure 2). A radar speed gun (BSG-1 Basic; Yupiteru) was used to measure the ball velocity of each pitch. Pitching performance was recorded every third pitch to prevent the camera system from overheating.

The strength of the medial elbow musculature in resisting valgus loading was determined in accordance with the procedure described elsewhere.^30^ It was measured for the pitching arm as the maximum voluntary isometric varus strength (MVIVS) of the medial elbow musculature at 3 time points during the pitching session: before pitching the first inning and after completing the fourth and seventh innings. Each participant was seated on the positioning chair of an isokinetic dynamometer system (Biodex System 4; Biodex Medical Systems), placing his dominant arm and forearm on the dynamometer attachment with the arm/leg support (830-154) and elbow/shoulder attachment (830-157) adjusted to his body size, so that the body posture resembled the standardized posture for shoulder internal rotation exercise, with the shoulder joint abducted to 90° and externally rotated to 60° and the elbow joint flexed to 90° (Figure 2). At this elbow joint angle, the valgus-varus axis of the elbow joint coincides with the internal-external rotation axis of the shoulder joint, so the shoulder internal rotation torque measured with the Biodex system can represent the net varus torque generated by the contractile forces of elbow musculature and the compressive forces of bony articulation around the valgus-varus axis when the UCL is not taut.^30^ To allow later quantitative examination of a widening of the medial joint space, that is, a physical sign indicative of valgus angulation, and the associated increased tension of the UCL (Figure 2), the width of the medial joint space between the trochlea of the humerus and the sublime tubercle of the ulna was monitored during the strength assessment with an ultrasound device (ArtUs EXT-1H; TELEMED Ultrasound Medical Systems) genlocked with the dynamometer system. After a few familiarization trials with slight postural alterations to the trunk and shoulder joint to maximize comfort and force production, the participants performed a ramp contraction up to 100% of their maximum voluntary isometric strength of the shoulder internal rotation while maintaining maximal voluntary contraction of the medial elbow musculature. The largest torque detected while the medial joint space was not widened beyond a threshold distance was recorded for each trial.^30^ The threshold distance was predetermined for each participant through a valgus stress test^30^: the participant was placed in a supine position with the shoulder joint externally rotated and abducted to 90°, the elbow joint flexed to 90°, and the elbow muscles fully relaxed. A 0.5-kg weight was then attached to the wrist, which generated an external valgus load of approximately 1.3 N·m to widen the medial joint space. The trial was repeated twice with a 2- to 3-minute rest interval, and an additional trial was conducted when the measured torque in the second trial was at least 10% higher than that of the first trial. The largest torque in the trials was taken as the MVIVS of the participant's elbow for that inning. Because the threshold for performing a third attempt was set at a 10% increase, the measured MVIVS may have underestimated true strength. In fact, the MVIVS measured in later innings appeared to increase by up to 9% (1-6 N·m) from the first inning's value in 10 participants. For these participants, we assumed that the largest MVIVS of all their measurements represented the first inning's MVIVS in order to obtain a better estimate for true strength during the first inning.

### Data Reduction

A markerless motion capture system (Theia3D Version 2023.1.0.3161; Theia Markerless) was used to calibrate the pitching space (calibration error, <1 mm), identify the pitcher's body on each video image during the motion as a system of 15 rigid body segments, and compute the position and orientation of each segment. The output file containing the raw position data and the 9 elements defining 3 × 3 orientation matrices for all body segments in the C3D format were processed using custom software developed on a programming platform (MATLAB R2022b; MathWorks). The raw data were smoothed with a fourth-order Butterworth filter at various cutoff frequencies determined for each variable (8-18 Hz [13-18 Hz for pitching arm]) using residual analysis,^40^ and each body segment was modeled as a rigid body having the inertial parameters for Japanese athletes.^1^ The orientation matrices were converted into Euler parameters before smoothing.

An inverse dynamics approach determined the motion-dependent valgus load imposed at the elbow in each pitch. For this analysis, the hand and ball were assumed to move together so that the system of ball and hand was considered as a rigid body. The computed joint resultant torque of the elbow joint of the pitching arm was decomposed into 3 orthogonal components^13^: flexion torque, pronation torque, and varus torque. The varus component of the resultant joint torque represented the net torque exerted on the forearm by the muscles, ligaments, and other joint structures that connect the forearm and arm at the elbow joint in response to the valgus load generated by pitching motion. Since the varus component of the resultant joint torque and the motion-dependent valgus load are action and reaction, the maximum value of the varus torque was recorded to represent the magnitude of valgus load for each pitch and used for subsequent analysis.

### Data Analysis

For each participant, the maximum, mean, and minimum values of maximum valgus loads recorded across the 5 pitches of each inning, together with the corresponding ball velocity, were extracted for statistical analysis. The Shapiro-Wilk test revealed that all variables were normally distributed across the participants, and, thus, the results are presented as means ± standard deviations. A linear mixed-effect model with inning as a fixed effect was used to examine within-pitcher changes over the innings in valgus load (across 7 innings), MVIVS (across 3 innings), and fastball velocity (across 7 innings). Paired *t* tests were used to compare the MVIVS measured before pitching the first inning and after completing the fourth and seventh innings to the largest valgus load in the first, fifth, and seventh innings, respectively. The level of significance was set at .05. Additionally, a linear mixed-effect model with random intercept and slope was used to describe between-pitcher variability in the within-pitcher effects on the inning's largest valgus load, MVIVS, and the difference between the two^43^ (strength deficit = valgus load − MVIVS).

## Results

All pitchers except 3 sidearm pitchers (mean arm angle, 9.8°± 7.3°) threw from a three-quarters delivery (mean arm angle, 34.3°± 10.3°). The pitch types thrown by the participants were 4-seam fastball (n = 28), slider (n = 21), curveball (n = 20), cutter (n = 19), changeup (n = 16), splitter/forkball (n = 13), 2-seam fastball (n = 5), and sinker (n = 2). Fastballs were pitched most frequently (58% ± 8.5% of all pitches), and the ratio of fastball count was not significantly changed over the innings (*P* > .05). The maximum fastball velocity of the participants ranged from 32.5 m/s to 39.7 m/s (mean, 36.8 ± 2.16 m/s or 82.4 ± 4.8 mph), and the inning's fastest velocity was not significantly changed over the 7 innings (*P* = .057) except in the fifth inning. The maximum velocity of the fifth inning (mean, 35.9 ± 2.27 m/s or 80.2 ± 5.1 mph) was significantly slower (*P* < .004) than those of the first (mean, 36.4 ± 1.92 m/s or 81.5 ± 4.3 mph), second (mean, 36.4 ± 2.01 m/s or 81.5 ± 4.5 mph), and third (mean, 36.6 ± 2.17 m/s or 81.2 ± 4.8 mph) innings.

**Figure 3.**
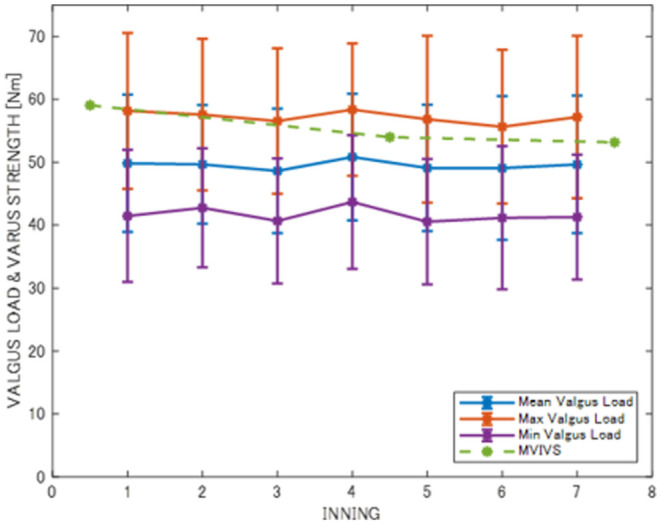
The medial elbow varus strength (maximum voluntary isometric varus strength [MVIVS]) and valgus loading on the pitcher's elbow over the 7 innings.

Participants’ maximum valgus load ranged from 41 N·m to 94 N·m (mean, 64.8 ± 13.7 N·m), whereas maximum MVIVS ranged from 40 N·m to 92 N·m (mean, 59.1 ± 13.4 N·m). Neither the largest valgus load of the inning (the mean across participants ranged from 55.4 to 58.6 N·m) nor the mean valgus load of the inning (the mean across participants ranged from 48.6 to 50.7 N·m) significantly changed across the innings (*P* > .437). On the other hand, the MVIVS measured before pitching the first inning (mean, 59.1 ± 13.4 N·m) was significantly decreased by 8.6% and 10.0% after completing the fourth (mean, 54.0 ± 15.2 N·m; *P* < .001) and seventh (mean, 53.2 ± 14.5 N·m; *P* < .001) innings, respectively (Figure 3). The MVIVS measured at the corresponding innings was significantly greater (*P* < .008) than the mean valgus load in the first inning (mean, 49.9 ± 11.1 N·m), but not different from the mean valgus loads in the fifth and seventh innings (mean, 49.2 ± 10.4 N·m and 49.7 ± 11.1 N·m, respectively), nor was it significantly different from the inning's largest valgus loads in all 3 innings (mean, 58.3 ± 12.9 N·m, 57.1 ± 13.8 N·m, and 57.2 ± 13.2 N·m in order).

**Figure 4.**
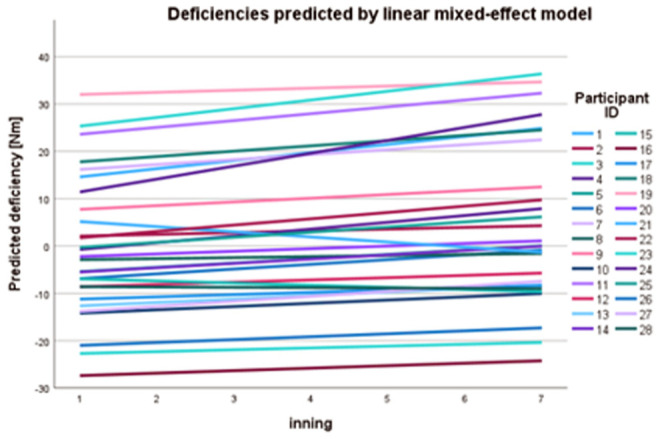
Strength deficits for all participants predicted by linear mixed-effect models. The strength deficit was defined by the difference between the inning's largest valgus load and the maximum voluntary isometric varus strength (MVIVS) of the inning (strength deficit = valgus load − MVIVS).

**Figure 5.**
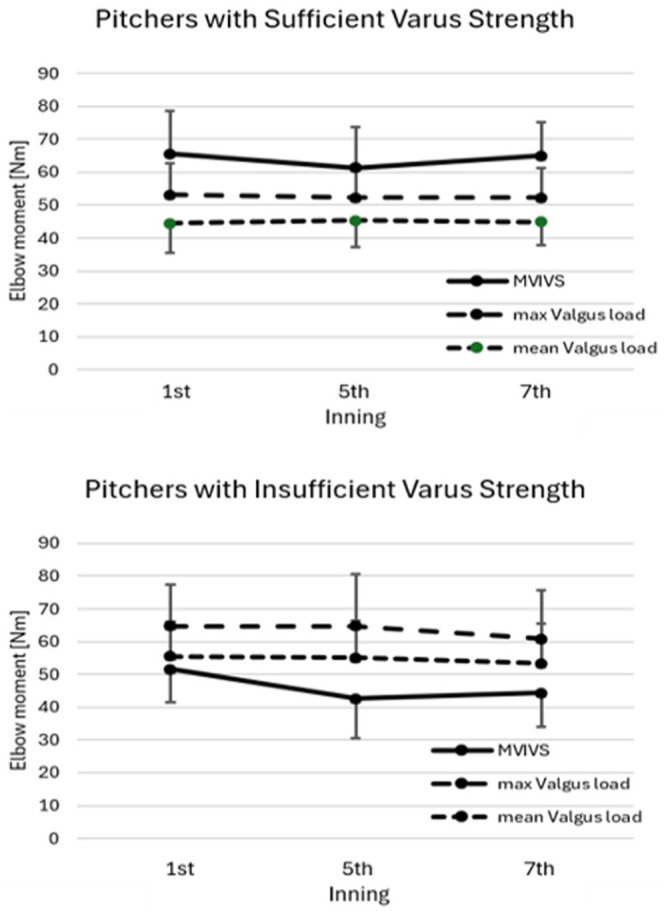
The medial elbow varus strength (maximum voluntary isometric varus strength [MVIVS]) and valgus loading on the pitcher's elbow of the pitchers with sufficient (top) and insufficient (bottom) varus strength to overcome the inning's largest valgus load. Four participants with a history of elbow pain (including one who had undergone ulnar collateral ligament reconstruction) had sufficient strength, and one other participant with a history of elbow pain had insufficient strength.

The predicted intercepts and slopes of the linear mixed-effect model for the varus strength ranged from 36 N·m to 87 N·m and −1.329 to −0.471, respectively. The corresponding values for the inning's largest valgus load ranged from 41 N·m to 82 N·m and −0.722 to 0.293, respectively. As a result of these interactions, the ranges for the predicted intercepts and slopes for strength deficit widened further, ranging from −28 N·m to 32 N·m and −1.136 to 1.708, respectively (Figure 4). In the first, fifth, and seventh innings, there were 8, 9, and 11 pitchers, respectively, whose mean valgus load for that inning exceeded the MVIVS measured at corresponding innings, and 13, 11, and 16 pitchers, respectively, whose strength deficit was positive (ie, the largest valgus load for that inning exceeded the MVIVS). The pitchers with insufficient muscular varus strength to overcome the inning's largest valgus load had lower MVIVS values in all 3 innings (mean, 51.5 ± 9.9 N·m, 42.5 ± 12.1 N·m, and 44.4 ± 10.3 N·m in order) and higher valgus loads in the first and fifth innings (mean, 64.7 ± 12.6 N·m and 64.7 ± 16.0 N·m, respectively) than those with sufficient strength (mean muscular varus strength, 65.6 ± 12.9 N·m, 61.4 ± 12.2 N·m, and 64.9 ± 10.3 N·m in order; mean valgus load, 53.0 ± 9.8 N·m and 52.2 ± 9.9 N·m for the first and fifth innings, respectively) (Figure 5).

## Discussion

This study was conducted to determine the effects of 105 pitches on the motion-dependent valgus load applied to the elbow joint and the strength of the medial elbow musculature that resists valgus loading. The results revealed that (1) the largest valgus load of the inning and the mean valgus load of the inning did not significantly change across the innings, (2) the MVIVS was reduced significantly after completing the fourth and seventh innings of pitching, and (3) the MVIVS was significantly greater than the mean valgus load in the first inning, but not in the later innings, nor was it significantly different from the inning's largest valgus loads in all 3 innings. These findings indicate that the pitcher's muscular varus strength weakens as the pitch count accumulates, and the pitchers are no longer able to generate a muscular varus moment sufficient to overcome the mean valgus load during pitching in the latter innings. Our hypotheses were supported. In addition, more than half of the pitchers lacked sufficient muscular varus strength to fully resist the inning's maximum valgus load by the seventh inning. Insufficient dynamic stabilizers to withstand valgus loading during pitching may open the inner elbow compartment^24^ and potentially causes elongation of the UCL that over time can lead to attenuation and failure.^34^ Therefore, our findings provide quantitative evidence suggesting that weakening of the active muscle restraint against valgus loading due to repetitive pitching places increased demands on the UCL in restraining the valgus angulation during pitching delivery.

This study revealed that the participants’ muscular varus strength decreased by a mean of 10% after 7 innings of 15 pitches, providing the first direct evidence that repeated pitching causes overall fatigue in the active muscle restraints against valgus loadings. Although several studies have reported the effect of repeated pitching on the strengths of forearm, elbow, and shoulder muscles, no studies have examined the weakening of the overall strength of the dynamic stabilizers’ ability to restrain the medial elbow against valgus loading. Pei-Hsi Chou et al^31^ focused on the strengths of elbow flexors and extensors, pronators, and shoulder muscles, and measured the isometric strength using a Biodex dynamometer before and after 110 pitches in 16 pitchers from the winning team of Taiwan's National High School Baseball Championship. Elbow flexor and extensor strengths decreased by means of 2.9% and 1.6%, respectively, and pronator strength decreased by 5.6%, while shoulder internal and external rotator strengths decreased even more, by means of 10.2% and 13.1%, respectively. Namiki et al^28^ measured the grip strength and forearm flexor strength of 31 collegiate baseball pitchers before and after pitching 15 pitches per inning for 7 innings. They reported that the grip strength and the strengths of wrist flexors, radial deviators, and ulnar deviators all decreased by a similar amount of 11% to 12%. Mullaney et al^26^ focused on the strength and fatigue of the middle and ring fingers’ flexors, hypothesizing that these were indicative of the strength and fatigue of the flexor digitorum superficialis and flexor carpi ulnaris muscles, and compared the strengths of the middle and ring fingers of 18 high school and collegiate baseball pitchers before and after 84 pitches (21 pitches per inning over 4 innings). The middle finger's flexor strength decreased by 21%, while the ring finger's flexor strength and grip strength decreased much less, 5% to 7%. Anatomically, the flexor carpi ulnaris muscle crosses the medial aspect of the elbow joint and ulnar side of the wrist joint and the flexor digitorum superficialis muscle crosses the medial aspect of the elbow, wrist, metacarpophalangeal, and proximal interphalangeal joints, so contraction of these muscles produces flexor and ulnar deviation moments at wrist and flexor moments at the metacarpophalangeal and interphalangeal joints in addition to the elbow varus moment. Therefore, it is not surprising that the findings of previous studies on grip strength,^28^ ulnar deviator and wrist flexor strengths,^28^ and middle and ring fingers’ flexion strengths^26^ are in line with our study and that the extent of the weakening of the dynamic elbow stabilizer's varus moment producing capability as a whole was within the range of strength declines of related muscle groups reported in the literature. The agreement between our results and the literature indicates that repeated baseball pitching has weakened individual muscle groups of pitching arm to varying degrees, resulting in overall fatigue across the dynamic stabilizer muscles and a 10% decrease in varus moment production capacity by the completion of 105 pitches.

Despite the fact that the medial elbow musculature was significantly weakened after repeated throws, neither the magnitude of the valgus load on the elbow joint nor the ball velocity declined over the 105 pitches. These observations are generally consistent with the literature. Escamilla et al^11^ examined kinetic and kinematic changes during pitching 15 pitches per innings for 7 to 9 innings (mean pitch count, 123 [8.2 innings]) in 10 collegiate baseball pitchers and reported that the ball velocity did not change significantly except in the last 2 innings pitched, and all kinematic and kinetic variables except trunk forward tilt angle did not change over the entire innings of pitch. Based on the observations, the authors noted that the pitching mechanics of the group of collegiate pitchers remained remarkably consistent. By measuring the valgus loading at the elbow and the EMG activity of the biceps brachii, triceps brachii, and flexor-pronator muscle mass during pitching of 60 to 110 pitches, van Trigt et al^37^ examined the effects of repeated pitches on the magnitude and variability of elbow valgus loads in 3 professional and 12 recreational pitchers. The results showed that within-pitcher variability in ball velocity, valgus loading, and EMG activity levels were not significantly associated with cumulative pitch count at the group level. The authors argued that this was primarily due to the high variability in the individual association among participants, indicating there was no uniform pattern in the changes in ball velocity and valgus loading over the repeated pitching across the participants. In the aforementioned study by Mullaney et al,^26^ ball velocity and elbow valgus loading were measured before and after 84 pitches in 18 high school and collegiate baseball pitchers, but no significant changes were observed across the innings. Okoroha et al^29^ reported contrary observations in 5 high school and 6 Division II collegiate baseball pitchers who pitched 15 pitches per inning for 6 innings, resulting in an approximately 2% decrease in ball velocity and an approximately 9% increase in valgus load (the percentages were estimated from Figures 2 and 3 in their study) over the 6 innings of pitching. Although there is some variability across the studies regarding the effect of repeated pitching on valgus loading, highly trained competitive baseball pitchers generally appear to have sufficient skill and stamina to consistently repeat the pitching mechanics over multiple innings of pitching, even when the force generating capability of some muscles is reduced by fatigue. On the other hand, given the mechanics of elbow injury, valgus loads that remain constant throughout multiple innings of pitching must be resisted by fatigued dynamic valgus stabilizer muscles in later innings, resulting in increased reliance on the UCL to withstand the valgus loads in latter innings of pitching. Based on these observations, we believe that proper conditioning and strengthening of the dynamic valgus stabilizers is critical in preventing UCL overload and reducing the risk of valgus overload injuries of the elbow.

**Figure 6.**
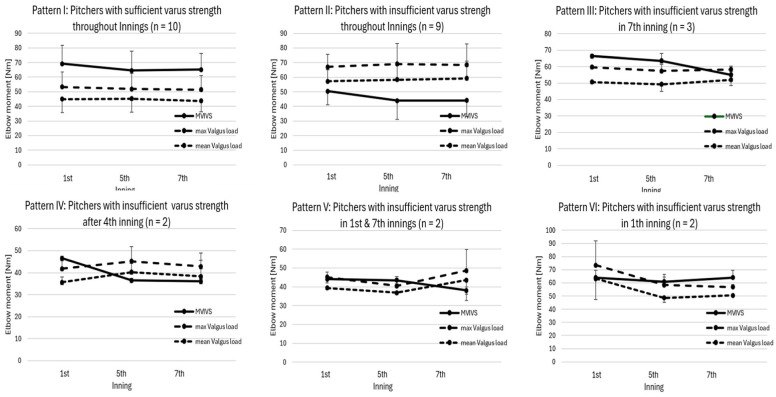
Individual differences in the response to repeated pitching observed in medial elbow varus strength (maximum voluntary isometric varus strength [MVIVS]) and valgus load on the pitcher's elbow. Overall, MVIVS decreased as the innings progressed, with an obvious drop occurring midway or late in the pitching innings. Valgus load remained constant throughout the 7 innings of pitching for the majority of pitchers (patterns I, II, and III). However, for some pitchers, valgus load increased (patterns IV and V) or decreased (pattern VI) as the inning progressed, exceeding muscular varus strength (MVIVS) in the later innings or the first inning, respectively.

At the group level, no significant differences were found between individual muscular varus strength and the corresponding inning’s largest valgus load throughout the 7 innings. However, individual responses to repeated pitching varied substantially, even more than indicated by between-pitcher variability in linear mixed-effect models in Figure 4. Comparison of MVIVS and largest valgus load in the first, fifth, and seventh innings revealed 6 development patterns of strength deficit (Figure 6): strength deficits were developed in 0 innings (pattern I), all innings (pattern II), only the seventh inning (pattern III), the fifth and seventh innings (pattern IV), the first and seventh innings (pattern V), and only the first inning (pattern VI). These differences are, in part, accounted for by individual differences in the development of muscular fatigue in pitcher's elbow dynamic valgus stabilizers: some pitchers showed a clear decline of >10% midway through the pitching innings (Figure 6, patterns II and IV), whereas others showed a similar level of decline at the end (Figure 6, patterns III and V). The differences in developmental patterns of strength deficit are also accounted for by individual differences in valgus loads across the innings: The majority of pitchers maintained a fairly constant valgus load throughout the 7 innings (Figure 6, patterns I, II, and III), but other pitchers showed a notable increase (Figure 6, patterns IV and V) or decrease (Figure 6, pattern VI) in motion-dependent valgus loads as the inning progressed. The difference in the patterns of change between MVIVS and valgus load across the innings led to individual variability in whether and when the dynamic valgus stabilizers alone were no longer able to fully resist valgus loads: pitchers demonstrating pattern VI, patterns III and IV, and pattern V failed to fully counteract valgus loads by the medial elbow musculature alone in the first inning, the later innings, and the first and seventh innings, respectively. Furthermore, because of the individual variance in MVIVS and its pattern of change across the innings, a valgus loading of 60 N·m, slightly greater than the mean value of the inning's largest valgus load in this study, can be considered within a safe range for pitchers demonstrating patterns I and VI, but would be an overload for pitchers demonstrating patterns II, IV, and V, as well as pitchers demonstrating pattern III in the seventh inning. These observations suggest that the magnitude of motion-dependent valgus load applied to the pitcher's elbow should not be considered an indicator of the ligamentous tension on the UCL. Based on the findings of substantial individual variability in muscular varus strength and its fatigue effects, we believe that an individualized approach is necessary to assess the risk of elbow valgus overload injury, and that quantification of loads and overloads requires measuring both the valgus loads during pitching and the strength of the medial elbow musculature that resists the valgus loading.

This study is limited by the following factors: First, we assumed that the first inning's true MVIVS should be the largest MVIVS of all measurements and adjusted for the 10 participants whose MVIVS measured in later innings was greater than the first inning's value. This adjustment increased the first-inning MVIVS from 57.5 ± 13.6 N·m without adjustment to 59.1 ± 13.4 N·m, and excluding adjusted participants increased the MVIVS to 58.0 ± 15.2 N·m, but neither the adjustment nor exclusion altered the significant differences obtained in the mixed-effect modeling (Table 1) or the *t* test between the MVIVS and the first inning's valgus load. These findings suggest that the main findings of this study were not sensitive to the adjustment made to the first inning's MVIVS. Second, the 120 Hz frame rate used in this study may not be sufficient to capture dynamic motion of rapid kinematic events, such as the ballistic movements of the fingers imparting spin to the ball. However, the maximum valgus load acting on the elbow was attained during the arm cocking phase or the transition period between the arm cocking and acceleration phases, and therefore the linear and angular motions of the pitching arm were still submaximal, making it unlikely to be at high risk for underestimation of peak values due to insufficient temporal resolution. Although the mechanics and ball velocity differ, the robustness of joint torque computation against reduced frame rates has been demonstrated in previous findings of similar overhead throwing,^15^ showing that peak varus torques (49, 46, and 59 N·m for 3 players) of quarterback passes (ball velocity: 21 ± 2 m/s) computed from marker-based motion capture data acquired at 200 Hz were nearly identical to the corresponding values (49, 44, and 59 N·m) computed from a frame-reduced version (67 Hz) of the same data set. Furthermore, our data, including the maximum valgus load (mean, 65 ± 13.7 N·m; range, 41-94 N·m) and external rotation angle of the shoulder joint at that instant (174.2°± 9.6°), fell within the range of corresponding values measured in similar populations using marker-based systems operated at higher sampling frequencies.^3,13,14,33,36^ The general agreement between the numeric results obtained in the present study and the existing literature indicates external validity. Additionally, to account for recent evidence that markerless motion capture systems similar to the one used in this study may underestimate varus torque during baseball pitching by approximately 10% compared to marker-based systems,^2^ we simulated a 10% to 15% increase in valgus loads obtained in our study and found that (1) the motion-dependent valgus load remained roughly constant throughout the 7 innings, (2) the maximum valgus loads were generally greater than the MVIVS throughout the innings, and (3) more pitchers failed to overcome the valgus load in the later innings. Whereas potential systematic errors in the determination of the valgus load might still lead to a question on the validity of the numeric results of the valgus load, they do not alter the main findings of the present study. These observations collectively suggest that the potential systematic errors associated with the present methods do not compromise the fundamental conclusions of the study. Third, all participants were members of a competitive collegiate baseball team, so the findings may not be applicable to Little League baseball pitchers and recreational-level baseball pitchers. Finally, the participants were asked to pitch in a simulated game with 15 pitches per inning for 7 innings; therefore, the findings and implications do not represent, nor do they predict, individual differences in the effects of repeatedly pitching a specific pitch type, or the cumulative effects of repeated pitching in multiple games over the course of several weeks or months during a baseball season. Further research is needed to explore the long-term effects of repetitive pitching on fatigue.

**Table 1:** Linear Mixed-Effect Models of MVIVS With and Without Adjustment*^*a*^*

	Intercept			Slope		
	Estimate	*P* Value	95% CI	Estimate	*P* Value	95% CI
No adjustment	58.1	<.001	52.7 to 63.5	−0.742	<.001	−1.15 to 0.33
Adjusted	59.9	<.001	54.7 to 64.9	−1.023	<.001	−1.33 to −0.71
Excluded (n = 18)	59.1	<.001	51.7 to 66.4	−1.310	<.001	−1.64 to −0.98

a MVIVS, maximum voluntary isometric varus strength.

## Conclusion

At the group level, pitchers’ medial elbow muscular varus strength decreased by a mean of 10% after 7 innings of 15 pitches, while motion-dependent valgus load remained fairly constant throughout the 7 innings, which may indicate increased reliance on the UCL to withstand the valgus loads in later innings. At the individual level, there were substantial differences in the patterns of change in MVIVS and valgus loads across innings, potentially resulting in individual variability in whether and when the dynamic stabilizers against valgus loading alone are no longer able to fully resist valgus loads. These observations suggest that medial elbow strength training should be incorporated into routine baseball practice to reduce reliance and forces on the UCL, and that risk assessment for elbow valgus overload injury should include measuring both the valgus loads during pitching and the medial elbow strength to resist valgus loading.
